# Evaluation of antifungal susceptibility in clinical isolates of the *Candida glabrata* complex

**DOI:** 10.1080/07853890.2021.1895583

**Published:** 2021-09-28

**Authors:** Filipa Jesus, Helena Barroso, Teresa Nascimento

**Affiliations:** a Instituto Universitário Egas Moniz (IUEM), Egas Moniz Cooperativa de Ensino Superior, Caparica, Almada, Portugal; b Centro de Investigação Interdisciplinar Egas Moniz (CiiEM), Egas Moniz Cooperativa de Ensino Superior, Caparica, Portugal; c Escola Superior de Saúde Egas Moniz (ESSEM), Egas Moniz Cooperativa de Ensino Superior, Caparica, Portugal

## Abstract

**Introduction:**

*Candida glabrata* is classified as an emerging threat due to its resistance profile to antifungal drugs. Associated to this, there is also the fact that recently, new species of *Candida* sp. phylogenetically related to *Candida glabrata* have been discovered: *Candida bracarensis* and *Candida nivariensis* [1]. Once that is only possible to identify these species through molecular methods [2], that identification represents a crucial step, since these species have been associated with a higher virulence and resistance to antifungals, in particular to the azole class [3], including the new extended-spectrum triazoles [4]. The aim of this study is to characterise *C. glabrata* clinical isolates from a culture collection.

**Materials and methods:**

Seventy clinical isolates from the “Micoteca IUEM” were used, presumedly classified as *Candida* sp. Their phenotypic identification was performed, and all isolates classified as *Candida glabrata* were subjected to molecular identification through the PCR technique followed by electrophoresis, to verify the presence of cryptic species. Susceptibility tests were performed using the disc diffusion method in order to evaluate the susceptibility of the complex to fluconazole and voriconazole.

**Results:**

Phenotypic identification showed that only 43 (61%) corresponded to *C. glabrata*. Molecular identification of these 43 isolates was carried out but led to inconclusive results. Susceptibility tests showed that one of the 43 samples of *C. glabrata* lost viability, 13 (31%) were sensitive to fluconazole, 12 (29%) were dose-dependent intermediates and 17 (40%) were resistant to fluconazole. Testing voriconazole, only 2 (5%) were resistant and the great majority, 40 (95%), was shown to be sensitive to voriconazole.

**Discussion and conclusions:**

This study showed that resistance to fluconazole is increasing and needs to be resolved quickly, since resistance in most cases was verified. However, voriconazole appears to be a good option for resistance to fluconazole, because it has been shown to be effective in the vast majority of strains resistant to fluconazole. One of the negative implications of this study is the fact that it is not possible to identify the users who have this resistance. Finally, it is important to highlight the need to produce new antifungal agents with different mechanisms of action, in addition to moderate and optimise the use of existing drugs.
